# In-Depth Comparative Assessment of Different Metallic Biomaterials in Simulated Body Fluid

**DOI:** 10.3390/ma14112774

**Published:** 2021-05-24

**Authors:** Radu Mirea, Andrei Tiberiu Cucuruz, Laurentiu Constantin Ceatra, Teodor Badea, Iuliana Biris, Elisa Popescu, Alexandru Paraschiv, Razvan Ene, Gabriela Sbarcea, Mihaiella Cretu

**Affiliations:** 1 Romanian Research and Development Institute for Gas Turbines—COMOTI, 220D Iuliu Maniu Blvd., 061126 Bucharest, Romania; laurentiu.ceatra@comoti.ro (L.C.C.); teodor.badea@comoti.ro (T.B.); alexandru.paraschiv@comoti.ro (A.P.); mihaela.cretu@comoti.ro (M.C.); 2 SC Cromatec Plus SRL, 1 Petre Ispirescu Street, 077167 Snagov, Romania; andrei.cucuruz@gmail.com; 3 Deptarment of Orthopedics and Traumatology, University Emergency Hospital, 169 Splaiul Independentei, 050098 Bucharest, Romania; ema.biris@gmail.com; 4 Bucharest Emergency Clinical Hospital, 8 Floreasca Street, 014461 Bucharest, Romania; elisa_zukie@yahoo.com; 5 Deptarment of Orthopedics and Traumatology, Carol Davila University of Medicine and Pharmacy, 8 Eroii Sanitari Blvd., 014464 Bucharest, Romania; 6 National Research and Development Institute for Electrical Engineering ICPE-CA, 030138 Bucharest, Romania; gabriela.sbarcea@icpe-ca.ro

**Keywords:** metallic biomaterials, ICP-MS, simulated body fluid, orthopedic implant, biomaterials characterization

## Abstract

Invitro experiments have been conducted on metallic biomaterials used for orthopedic implants in order to determine their behavior when immersed in simulated body fluid (SBF). Thus, 3Ti-based metallic biomaterial samples already available on the marked were purchased and immersed in simulated blood plasma, and kept at 37 °C for 4 months. In-depth characterization consisted of a wide series of structural characterizations of both the samples and SBF. Sample analysis consisted of the following: optical (OM) and scanning electron microscopy (SEM) in order to establish the surface and deep corrosion, mass gain/loss assessment for determining the metallic ions loss and/or protective layer formation, and X-ray diffraction in order to establish if and what kind of layers are formed. SBF analysis consisted of using inductively coupled plasma mass spectroscopy (ICP-MS) in order to establish if and/or how many metallic ions have dissociated from the metallic samples into the SBF, and measurements of pH and electrical conductivity. The key findings of the research are as follows: during the four months while kept in SBF, the samples show surface corrosion degradation and protective layer generation. Also, the amount of metallic ions dissociated into the SBF is making them suitable for use. Taking into account that it is highly improbable for such a large area of metal as the one considered within this work to be exposed to real body fluids and that all the samples have developed protective oxide films, the overall conclusion is that they are appropriate for implant use.

## 1. Introduction

The demand for metallic materials in medical devices is large. Metals and alloys are widely used as biomedical materials and some of their particular features, such as toughness, elasticity, rigidity and sometimes electrical conductivity, make them suitable for such applications. Titanium and titanium alloys are attractive structural materials due to their high strength, low density and excellent corrosion resistance. The excellent corrosion resistance of titanium alloys results from the formation of very stable, continuous, very adherent and protective oxide films on metal surfaces. Because titanium is highly reactive and has an extremely high affinity for oxygen, these beneficial surface oxide films form spontaneously and instantly when fresh metal surfaces are exposed to air and/or aqueous media [1]. It has been observed that the detachment of wear particles is one of the main problems associated to total hip replacement and in order to overcome or minimize this problem alternative bearings have been developed. However, material release from implants has been reported. For example, increased concentrations of Co and Cr were found in the blood, serum and urine [2].

**Figure 1:**
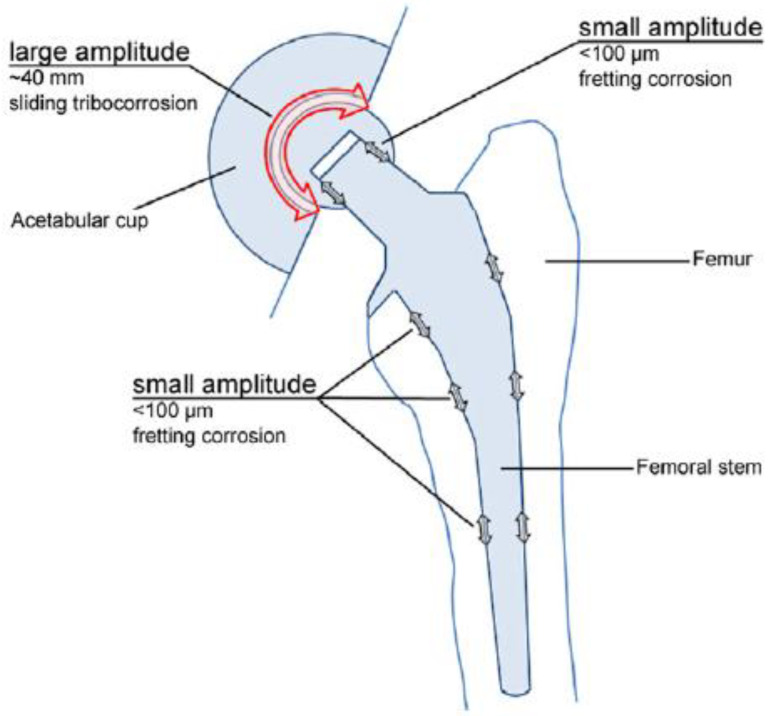
Total hip joint replacement prosthesis [3]. Reprinted with permission from Margareth R.C. Marques. Global Science and Standards U.S. Pharmacopeia.

In the particular case of orthopedic implants, micro-motions are known to occur at the points of fixation, while corrosion is caused by the body fluids, which contain various inorganic and organic molecules (see Figure 1) [3].

As the counter body moves on, the de-passivated surface area re-oxidizes, a process involving a change transfer reaction at the interface, which yields dissolved metal ions and a solid oxide [3]. Thus, the main aim of this paper is to assess the following aspects in case of implant surface damage: the re-formation of passivated oxide films and the amount of metallic ions released into the SBF. The SBF method as the invitro indicator was widely accepted by researchers immediately after its invention [4]. 

The first requirement of any material to be placed in the human body is that it should be biocompatible and not cause any adverse reactions. Corrosion and surface oxide film dissolution are the two mechanisms that introduce additional ions into the body. The extensive release of ions from implants can result in adverse biological reactions. Corrosion is the first consideration for a material of any type that is to be used in the body, because metal ion release takes place mainly due to the corrosion of surgical implants. The two main corrosion types that often appear are spot and pitting corrosion, as described in [4,5,6].

Titanium alloys must have high biocompatibility, good corrosion resistance and excellent mechanical properties for the use in fields such orthodontics and orthopedics. Some titanium alloys have received more attention as biomaterials due to their added improvements. The influence of alloying elements in titanium alloys contributes to a wide range of different micro-structural and mechanical properties. Thus, the alloying elements have been divided into the following three distinct categories: α stabilizers: C, N_2_, O_2_, Al having the main role of extending the hexagonal α-phase field to elevated temperatures; β stabilizers: V, Nb, Mo, Co, Cr, Ni, Cu, W, etc., who shift the β-phase field to lower temperatures; and neutral elements: Zr, Sn, Hf, Ge [7,8]. In order to be accepted as biomaterials, titanium alloys must meet several demands and one of the most important ones is their low toxicity, meaning they should not release metallic ions into the body fluids, not even when the protective oxide layer is damaged.

Ti-based materials combined with Co and Cr are highly compatible [9,10,11], and are widely used as orthopedic implant materials in clinical practices such as hip joint and knee replacement due to their superior mechanical properties, good wear, and corrosion resistance [12]. The biocompatibility of Ti-based materials combined with Co and Cr is closely related to its excellent corrosion resistance due to the presence of an extremely thin passive oxide film that spontaneously forms on the alloy surface [12]. It was found that the surface oxide film of Co–Cr inhibits the dissolution of metal ions but is not always stable in the human body. Hanawa et al. characterized the surface oxide films formed on Co and Cr alloys during immersion in various biological environments [13,14].

Previous works that have assessed the corrosion of Ti-based biomaterials were using different techniques. Thus, in paper [15], Ti-based biomaterials have been tested in SBF but corrosion has been assessed along with the tribological properties of the material. The testing procedure consisted of an electrochemical assessment if the corrosion and key findings were that the warm-rolling manufacturing technique of TiNbZr biomaterials allows improved characteristics in terms of corrosion resistance within SBF. In [16], a sterile immersion fluid having high glucose concentration was used and the samples were kept immersed for two weeks, and the SBF was changed every 3 days. The testing procedure consisted of polishing the surface and then using a ball-on-disc wear device with mechanical contact between the sample and the measuring device. The key findings were as follows: a spontaneous layer of hydroxyapatite was formed on the sample’s surface and the corrosion rate was accelerated when immersed in SBF. Ref [17] presents advanced microscopy (SE and TEM) for assessing the localized oxidation of TiNi-based biomaterials that were immersed in SBF. The testing procedure implied static immersion, and following a sampling protocol, characterization has been made. Surface analysis has been used for assessing the oxidation and the key findings consisted of the localization of oxide particles on the sample’s surface.

The work conducted within this research deals the characterization of both metallic biomaterials and SBF solution by using scanning electronic microscopy, optical microscopy, XRD, and inductively coupled plasma mass spectroscopy (ICP-MS) [18,19]. The novelty of the paper is the assessment of, firstly, the appearance of surface corrosion, secondly the metallic ions dissociation, and thirdly the appearance of a spontaneous protective layer on the exposed surface.

## 2. Materials and Methods

In order to carry out the research and having in mind the abovementioned testing protocols [15,16,17], 3Ti-based biomaterials consisting of sample 1—CoCrTiNi hummers plate, sample 2—CoNiFeTi femoral rod, sample 3—NiFeTi clavicle platesthat have been procured from the market (hummers and clavicle plate from Aysam Ortopedi & Tibbi Aletler—Turkey and tibial rod from Austofix Australian Orthopaedic Fixation Pty. Ltd. (North Plympton, Australia) and prepared for testing. The approach consisted of assessing the following aspects: firstly, the appearance of surface corrosion, secondly the metallic ions dissociation and thirdly the appearance of spontaneous protective layer on the exposed surface.

Even though the metallic samples used within the current paper had protective films on their surface, sample preparation removed it before inserting the samples in SBF. The aim of this procedure consisted of assessing the amount of metallic ions dissociated within the SBF in case of damaging the protective surface of the metallic biomaterial used as the implant. Also, a calculus has been made in order to determine the amount of metallic ions dissociated from 1 mm^2^ of exposed metal. The samples were polished by using a rotating polishing machine (Metkon, Bursa, Turkey) until its roughness dropped below 0.05 µm (N2 class). The used polishing paper had a roughness varying from 240 to 1200 set by Federation of European Producers of Abrasives and after that the sample has been surfaced using diamond powder suspension of 3 and 1 µm, respectively. The exposed surface has been accurately measured by using Atos Compact Scan 5M laser-based measuring equipment (GOM GMBH, Düsseldorf, Germany) and the samples were weighted to 4 decimals. Then the metallic samples were immersed in an SBF solution for assessing the dissociation of the metallic ions from the sample into SBF and kept to a constant 37 °C temperature in a thermal bath and in relative motion related to the SBF at a frequency of 60 movements/minute on a distance of ±1 cm right/left in order to mimic the relative motion of body fluid against an implant. Each of the three abovementioned samples have been tested and characterized according to the following protocol: 0 h (initial), 24 h, 72 h, 168 h, 336 h, 672 h, 1344 h, 1920 h and 2760 h, thus resulting in 24 sample collections and analyses. 

Taking into account that even a small amount of metallic ion release into the body could be very aggressive due to their migration and accumulation in different organs, sometimes far away from the point of release, it is very important to quantify the amount of ion release in various environments, thus ICP-MS investigations were conducted [20]. Table 1 shows the maximum accepted concentrations of different metallic ions in human body.

### 2.1. Method for Preparing the SBF

An SBF solution was prepared as shown in [24], being an updated version of simulated body fluid submitted in 2003 with detailed instructions for its preparation to the Technical Committee ISO/TC 150 of International Organization for Standardization as a solution for in vitro measurement for implant materials. Table 2 shows the concentrations of reagents to be used for 1 L of SBF. The SBF was prepared in the lab by mixing extra-pure substances procured from the market from WWR Chemicals. The sample was immersed in the prepared SBF in an Erlenmeyer polypropylene (PP) recipient. The recipient was immersed in a thermostatic bath endowed with a movable device allowing a frequency of 60 movements/minute on a distance of ±1 cm right/left in order to mimic the relative motion of body fluid against an implant. By using a PP wire, it was ensured that the sample does not come in contact with the recipient, being kept immersed in SBF.

### 2.2. Sample Characterization

The metallic samples were assessed and characterized as described below:

1. ICP-OES characterization was performed on the samples in order to determine their exact metallic composition. ICP-OES is a specifically dedicated analytical method for determining large concentrations of metallic components within a given liquid sample;

The method has been used for determining the composition of the metallic biomaterials studied within the paper and the results are shown in Table 3.

2. Optical microscopy (OM) has been used to analyze the surface of the metallic biomaterials in order to highlight corrosion types (pitting, spotting, etc.). Thus, for surface modifications assessment, Axio Vert.A1 Mat metallographic microscope (Karl Zeiss AG, Oberkochen, Germany) was used enabling a magnification 50X and normal light;
3. Scanning electron microscopy (SEM) was made by using FEI Inspect F50 SEM microscope (FEI (today: Thermo Fisher Scientific), Brno, Czech Republic) for surface assessment, allowing a magnification of 4000X. The method has been used to emphasize deep corrosion and surface transformations of the metallic samples [25];
4. Mass loss/gain aiming to determine firstly the corrosion rate and secondly, correlated the formation of protective layers with XRD;
5. XRD analysis used to establish if/what kind of oxides have formed on sample’s surface. Bruker D8Discover diffract-meter (Billerica, MA, USA) was used. The diffract-meter settings were as follows: primary optics uses Cu tube (λ = 1.540598 Å) and Göebel mirror while secondary optics uses 1D Lynx Eye detector (Bruker, Billerica, MA, USA). The plots have been recorded at 0.04° angle and 1 s/step scanning speed. They have been indexed using ICDD Release 2015 database (2015 release, International Center for Diffraction Data, Newtown Square, PA, USA).

### 2.3. SBF Characterization

In case of metallic ions release, the SBF composition must change accordingly, so in order to establish this aspect, the following characterizations were performed on the SBF:

1. The pH and electrical conductivity characterizations were performed in order to determine the redox phenomena that occur and the variation in ions within the SBF. A Mettler-Toledo pH/conduct-meter (Mettler-Toledo, Greifensee, Switzerland) was used;
2. Inductively coupled plasma mass spectrometry (ICP-MS) [9] was used for determining the amount of metallic ions dissociated within the SBF. Dirac Elan II ICP-MS spectrometer (Perkin-Elmer, Toronto, ON, Canada) was used.

## 3. Results and Discussions 

All the samples have been tested from a structural point of view in order to assess both their degradation and stability while immersed in the above defined SBF. 

Thus, the samples have been firstly weighed, measured and pictures have been made. Also, the SBF was characterized in terms of metallic ions dispersed within it. These initial measurements offered the ground base of the experiments.

Table 4 presents the initial characteristics of the metallic samples, including mass, exposed surface and optical state.

### 3.1. OM Assessment

This assessment aims to highlight the surface corrosion that may occur during metal exposure to SBF. The measurements have been performed by using a 50X magnification in normal light.

**Figure 2:**
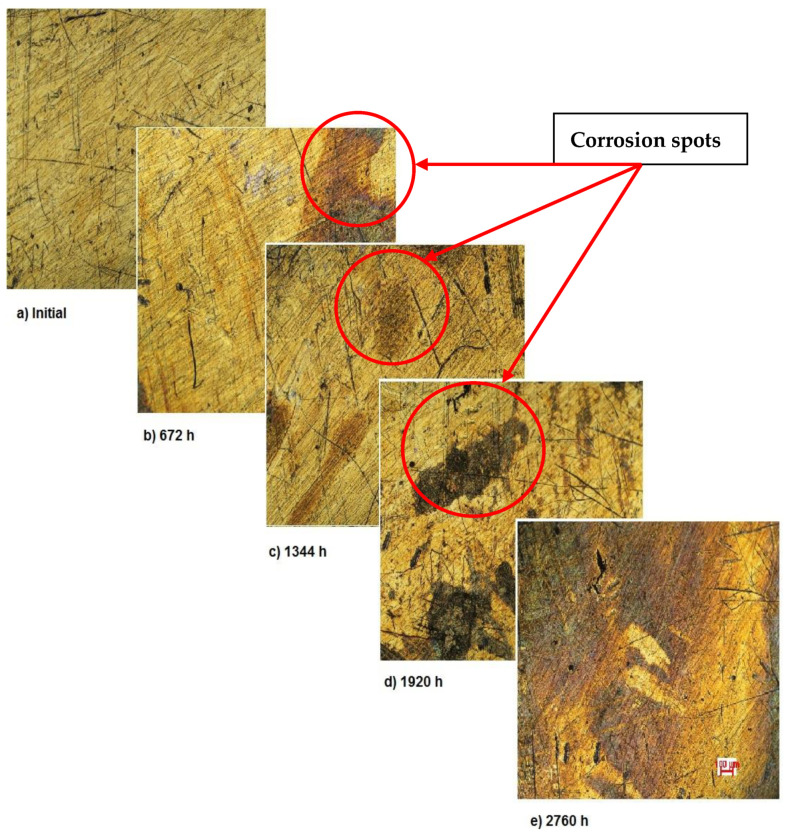
OM assessment of sample 1.

Figure 2 shows the OM assessment of the exposed surface of sample 1—CoCrTiNi hummers plate. As it can be seen, the sample is initially clean and does not show any dark spots. When exposed to the SBF, as time passes, even in the first month of exposure, dark spots of corrosion occur, and after the fourth month of exposure, the corrosion is generalized on the entire surface of the sample. The yellow color of the sample may be due to the formation of titanium oxide (NiTiO_3_) (as it can be seen in figures, representing the XRD plot for this sample).

**Figure 3:**
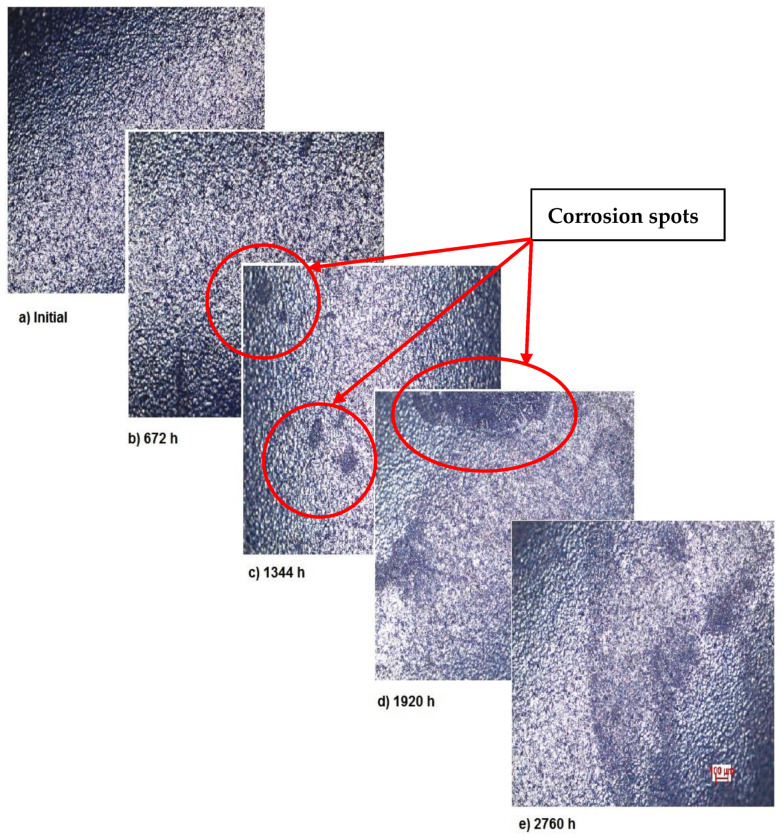
OM assessment of sample 2.

The OM assessment has been performed for sample 2 also—CoNiFeTi femoral rod—in order to determine its surface transformation during the 2760 h exposure to SBF. As it can be seen in Figure 3, some spots looking like corrosion appear starting with the second month of exposure and develop until the fourth month. Unlike the first sample, this one’s color is not yellow. According to figures representing the XRD for this sample, a protective layer of Ni_2_Ti occurs on the surface. 

**Figure 4:**
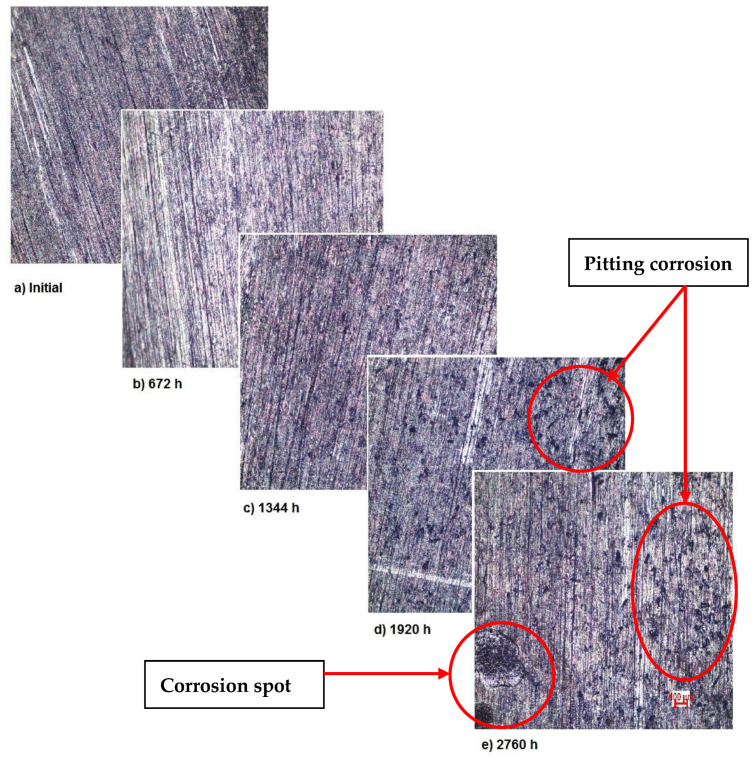
OM assessment of sample 3.

Also, for sample 3—NiFeTi clavicle plate—the OM assessment was made in order to determine the exposed surface corrosion of the metallic biomaterial. As shown in Figure 4, after the second month of exposure, pitting corrosion occurs on the sample’s surface, which develops over time and in the fourth month also corrosion spots appear on the surface. The sample’s color is given by the iron oxide (Fe_2_O_3_) that forms on its surface.

As demonstrated by the OM assessment, each metallic biomaterial develops surface corrosion during its exposure to SBF as follows: the first two samples develop spot corrosion and the third one pitting corrosion. Also, the samples develop protective layers on their surfaces as a response to the removal of the initial protective layer, as it can be observed in the figures representing XRD plots. The moment and type of corrosion varies according to each biomaterial’s inner structure and composition, but the main conclusion highlighted by this assessment is that whenever exposed metal is in contact with SBF, surface corrosion will definitely occur sooner or later.

The surface corrosion may occur under the influence of the acidic environment of the SBF (mainly due to HCl).

### 3.2. SEM Assessment

This type of assessment is aiming to investigate, even deeper, the corrosion that occurs on the surface. A magnification of 4000X was considered enough in this case and that is why the SEM assessment was performed by the means of this magnification. The covered area was 80 µm × 80 µm.

As in the case of OM, the analysis was performed on the exposed surface, aiming to highlight the formation of the protective layer as a replacement for the one removed. 

**Figure 5:**
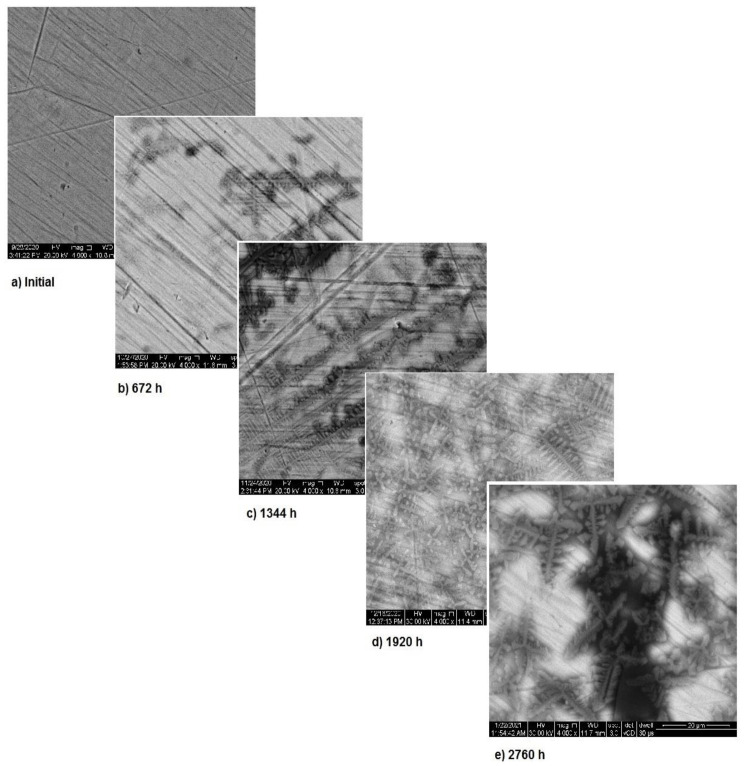
SEM assessment of sample 1.

Figure 5 presents the surface modifications implied by the corrosion for sample 1. As mentioned above, the corrosion appears during the first month of exposure and starts from the fine scratches on the metal surface. Over time, the surface corrosion spreads on the entire surface, thus showing the corrosion type as in Figure 2. However, the surface transformations highlight the formation of a new protective layer. Image analysis indicates the occurrence of corrosion starting from the first month of immersion and corrosion stopping after the third month. This is a strong indicator of the material’s stability.

**Figure 6:**
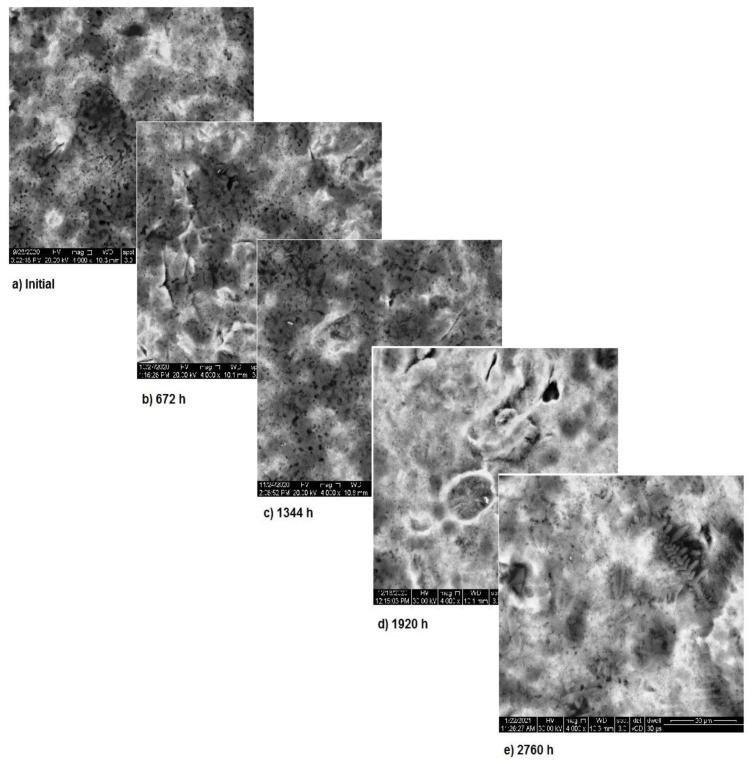
SEM assessment of sample 2.

The SEM assessment of sample 2 is shown in Figure 6 and highlights the more stable structure of this sample. As it can be seen, in the first two months of exposure, the sample’s surface is almost unaffected. Nevertheless, starting from month 3, surface modifications appear due to the corrosion and thus, spots form. In the first two months, the corrosion processes takes place at the surface of the sample and after that these processes come to an end, while the entire surface is covered with a new layer of protective material. Image analysis highlights the corrosion in spots and only limited pitting corrosion. 

**Figure 7:**
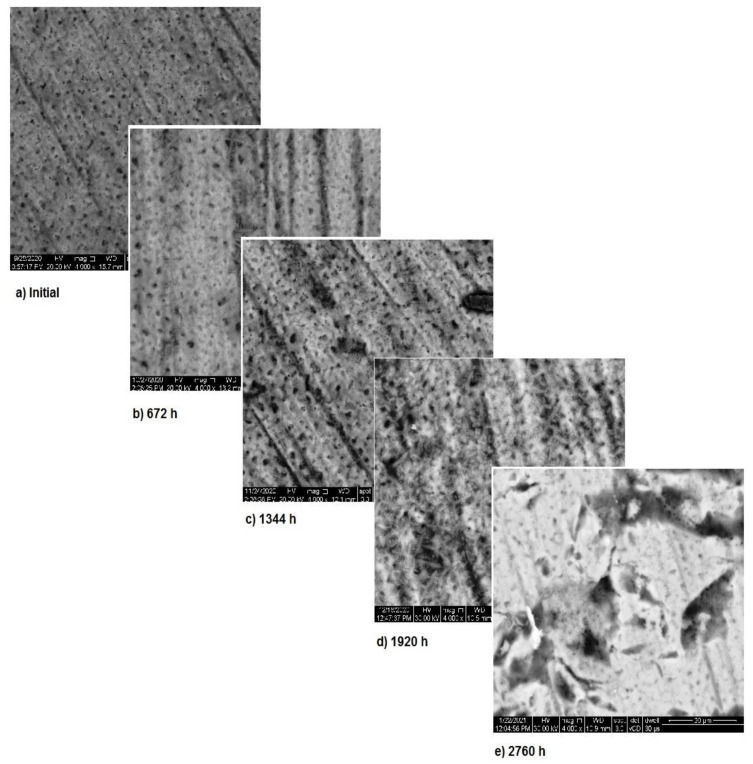
SEM assessment of sample 3.

In Figure 7, the surface corrosion of sample 3 is shown. In the first three months, pitting corrosion occurs and during the fourth month the surface is covered with a protective layer that covers the entire area. The images, correlated with the variation in the metallic ion concentration, pH and electrical conductivity, highlight the formation of the protective layer on the exposed surface.

Once again, the conclusion drawn from “Section 3.1—OM assessment” is that each metallic biomaterial develops surface corrosion during its exposure to SBF and the type of corrosion varies according to the material composition, is strengthened by SEM assessment. Furthermore, SEM assessment allows to understand how the corrosion process takes place and also allows the assessment of the protective layer.

### 3.3. XRDAnalysis

XRD is usually used in materials science for determining the crystallographic structure of a material, being one of the most used techniques in the field. It uses X-ray to irradiate the material. Firstly, an incident X-ray is sent towards the material, and the reflected X-rays are measured in terms of intensities and angles. 

Within this paper, X-ray diffraction (XRD) was used to establish if/what kind of oxides have formed on the sample’s surface. As stated before, a D8 Discover diffract-meter was used for sample analysis.

**Figure 8:**
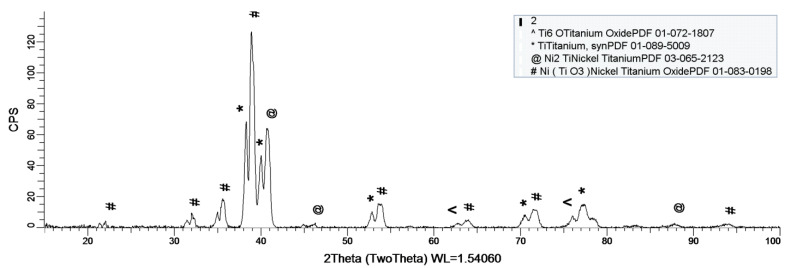
XRD plot for sample 1.

**Figure 9:**
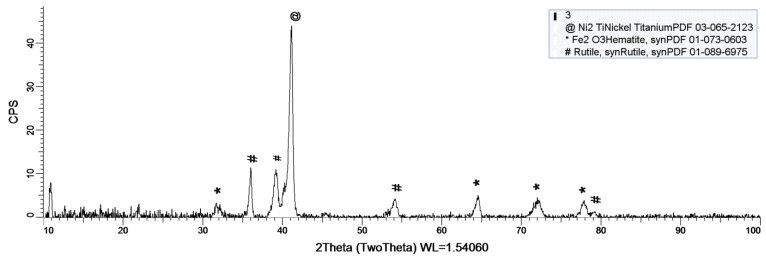
XRD plot for sample 2.

**Figure 10:**
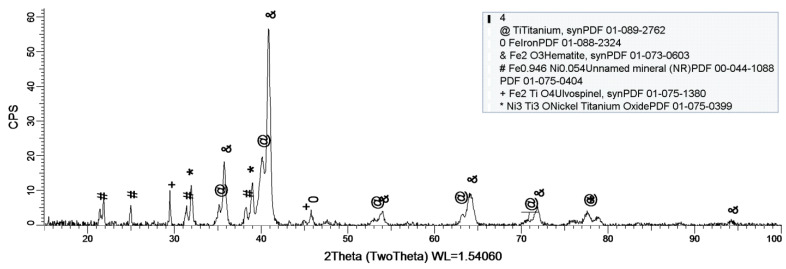
XRD plot for sample 3.

Figure 8, Figure 9 and Figure 10 show the formation of a protective layer during the sample’s exposure to SBF. Based on each sample’s composition, different layers form on the surface. 

As it can be observed in Figure 8, nickel-titanium oxide (NiTiO_3_) forms on the surface as the sample is immersed in the SBF. The oxide is well represented on the sample’s surface, leading to the conclusion that it takes a while, but after its formation the oxide layer offers a protective interface between the SBF and metallic implant.

Sample 2 provides a protective layer made out of Ti and Ni (Ni_2_Ti), which is actually an alloy. Indeed, the iron within the sample forms an oxide layer Fe_2_O_3_, but it is negligible.

Figure 10 shows the formation of several oxides on the sample’s surface, but the most important one is Fe_2_O_3_. Unlike the previous sample (sample 2), this one does not contain Co, thus allowing Fe to oxidize to its most stable form Fe_2_O_3._

As a general discussion related to XRD analysis, the main conclusion is that the sample’s composition drastically influences the formation of a protective layer on the surface. Thus, in the case of sample 1, the CoCrTi-based alloy is very stable, and the alloy made of Ni and Ti oxidizes until its most stable form, NiTiO_3_. In the case of sample 2, the missing Cr and the existence of Fe determine the formation of the Ni_2_Ti layer, which is actually an alloy. It is to be mentioned, that due to Co, the Fe within the structure does not oxidize. Sample 3 lacks the Co, thus Fe is able to oxidize, forming the layer on the entire surface of the sample.

### 3.4. ICP-MS Assessment

One of the most important issues tackled by using this technique is the quantitative assessment of metallic ions dissociated within the SBF. Therefore, mass spectroscopy was used to determine the precise amount of metallic ions that have been released into the SBF by the metallic biomaterials. Having in mind the determined composition of the samples, the following metallic ions have been determined. The procedure was described in 2.3 and consisted of analyzing the SBF solution. 

**Figure 11:**
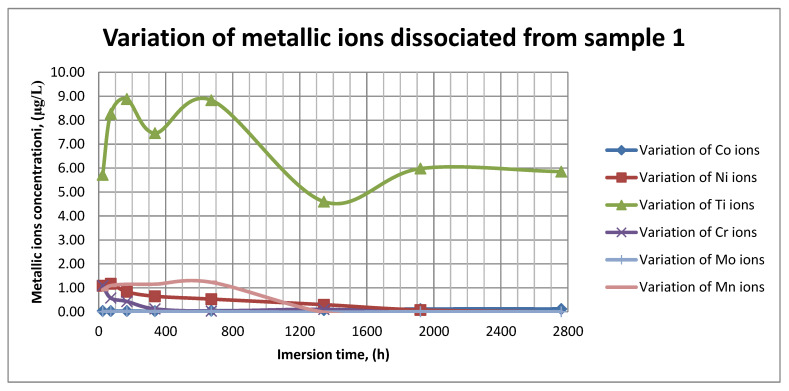
Variation in metallic ions dissociated from sample 1.

**Figure 12:**
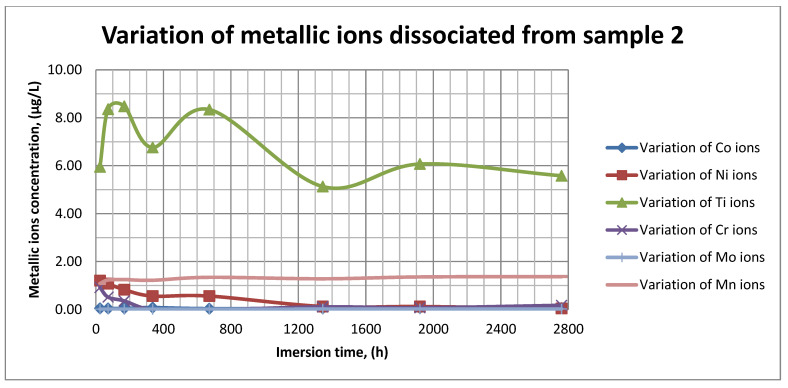
Variation in metallic ions dissociated from sample 2.

**Figure 13:**
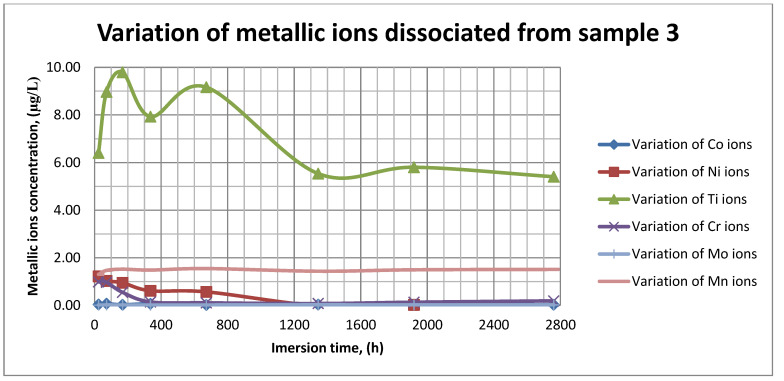
Variation in metallic ions dissociated from sample 3.

The plots within Figure 11, Figure 12 and Figure 13 show the variation in the main metallic ions dissociated from the metallic sample into the SBF solution.

As it can be seen in Figure 11, Figure 12 and Figure 13, some of the metallic ions (mainly Ti) are released and/or consumed over time. The most probable hypothesis is that this behavior may be the result of the formation of protective oxide on the sample’s surface. It is well known that most of the metallic biomaterials rapidly form protective oxide layers when scratched. Nevertheless, the maximum amount of released metallic ions is still way lower that the amounts supported by the human body, as presented in Table 1. Thus, the released amount of Ti ions ranges from 5–10 µg/g, while the maximum allowed amount is 800 µg/day, so roughly 100 µg/L, and the concentration of Cr ions dissociated is roughly 1µg/L, while the maximum accepted amount is 28 mg/L, etc. 

### 3.5. pH, Electrical Conductivity and Mass Variation Assessment

The formation of the protective layers were further investigated by mass variation, pH and electrical conductivity measurements as follows: mass variation for metallic materials used to assess the layers formation, and metallic ions release rate, pH and electrical conductivity for the SBF solutions in order to establish the presence of the metallic ions.

Table 5 shows the metallic ions release rate from each sample and their corrosion rate. The values have been calculated using the following formula:

(1) $$c_{i}=\frac{m_{i,\;sol}}{m_{i,sample}\times S\times 16}$$

where *c_i_*—ion concentration, *m_i,sol_*—ion mass in solution, *m_i,sample_*—ion mass in sample, *S*—exposed surface, and 16—no. of weeks of exposure.

The corrosion rate has been calculated by using the following formula:

(2) $$C_{r}=\frac{m_{m}}{m_{i}\times S\times 16}$$

where *m_m_*—measured mass of the sample, *m_i_*—initial mass of the sample, *S*—exposed surface, and 16—no. of weeks of exposure.

The corrosion rate is almost insignificant (×10^−5^) since its measure is grams over mm^2^ and week. Most of the implant materials are kept within the body for a maximum of 2 years (almost 100 weeks in very bad cases), and the overall corrosion rate can be declared as 4.3%. Also, the amounts of individual metallic ions is insignificant since the measured amounts range from ×10^−6^to × 10^−4^mg/L, and the allowed are ×10^1^mg/L. The more important aspect is the release rate of Mn, but as Figure 11, Figure 12 and Figure 13 show, this release is only in the first 100 h of exposure, then the trend is constant and it can be considered that Mn turns passive after 3 days.

**Figure 14:**
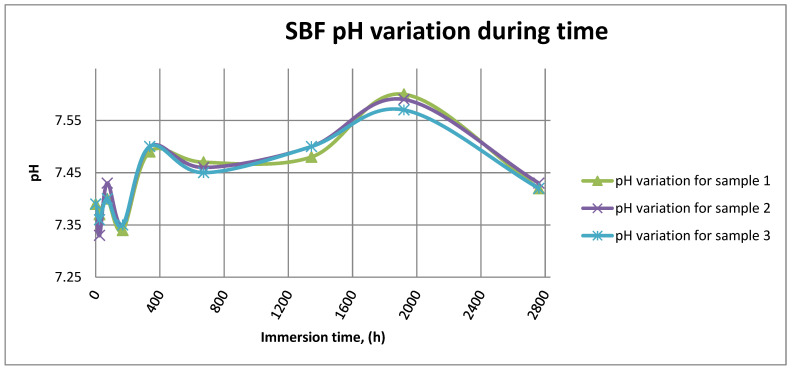
SBF pH variation for all samples.

Figure 14 shows the variation in pH for SBF in the case of all three samples.

**Figure 15:**
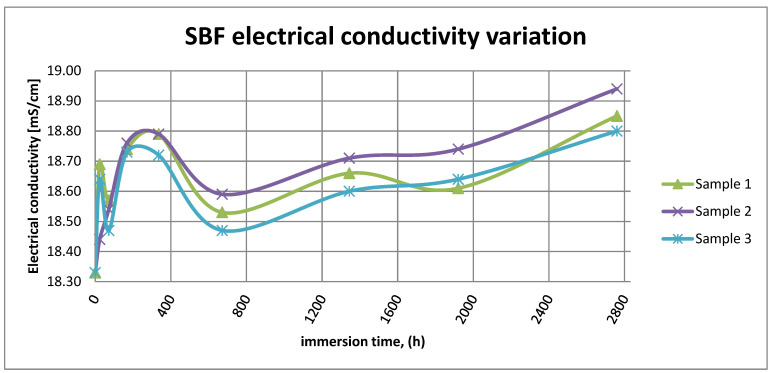
SBF electrical conductivity variation.

Figure 15 shows the variation in electrical conductivity of SBF in the case of all three samples.

As it can be observed in Figure 14 and Figure 15, the variation in the pH and electrical conductivity are closely interdependent. Thus, in the first two weeks of exposure, the SBF pH varies allot while the electrical conductivity increases. During this period, the formation of a protective layer on the surface occurs. Redox phenomena within the first two weeks of exposure are intense, as seen in Figure 14, and the concentration (µg/L) of metallic ions is increased, as can be observed in Figure 11, Figure 12 and Figure 13.

Furthermore, this trend continues during the second two weeks of exposure when the redox phenomena are still present but, as Figure 11, Figure 12 and Figure 13 show, the concentration of metallic ions decreases while the protective layers are forming, correlated with a continuous increase in the electrical conductivity and pH.

After the first month of exposure, the amplitude of redox phenomena diminishes as the protective oxide layer is formed on the sample’s surface.

## 4. Conclusions

- 3Ti-based biomaterials have been characterized within this paper in terms of surface corrosion, metallic ions dissociation and formation of spontaneous protective layers while immersed in SBF, using advanced investigation techniques aiming to highlight even more the biocompatibility of these materials;
- The type of corrosion that occurs was different (corrosion spots, pitting and/or mixture) as well the moment they appear. This fact can be attributed to the internal structure of the biomaterial as well as its composition. The corrosion types and formation mechanisms have been assessed in-depth by optical and scanning electron microscopy, proving that microscopy can be a very useful “instrument” in material characterization. Furthermore, corrosion development was finally stopped by the formation of a protective layer on the materials surface;
- The concentration of metallic ions dissociated from the biomaterial into the SBF solution was assessed by using the ICP-MS technique, which highlights that it varies during SBF solution exposure and the main conclusion is that those ions are forming a protective layer on the sample’s surface. This is mainly due to their reactivity and may lead to the passivation of the exposed surface, thus minimizing the number of ions that can dissociate in real body fluid. Moreover, given the obtained results, it is clear that the assessed implants are safe to be used since the levels of metallic ions that dissociate variation are 1.000 to 10.000 times less than the maximum accepted levels;
- Even though the material has a large area in direct contact with SBF, a new protective layer (oxide as in the case of sample 3 and alloy as in the case of sample 2) is formed relatively soon, as highlighted by the XRD assessment;
- The testing protocol and the equipment used within this paper are complementary to the already used ones for assessing the behavior and corrosion of Ti-based biomaterials in SBF.

## Figures and Tables

**Table 1:** Biological effects of metallic ion accumulation in the human body.

Metal	Effect
Nickel (Ni)	It is the main cause of contact dermatitis. The main biological parameter is the amount of metal released on the skin during direct contact and exposure to human sweat. The limit is 0.5 mg/(cm^2^ × week) of which an insignificant amount of Ni-sensitive subjects will react. It has toxic effect by creating cellular lesion and large cellular cultures. It is very dangerous for bones and tissues, although less dangerous than Co or V and it has cancer potency. The normal level of Ni in blood is 5 mg/L [21].
Titanium	There is no known biological role for titanium. There is a detectable amount of titanium in the human body and it has been estimated that we take in about 0.8 mg/day, but most passes through us without being adsorbed. It is not a poison metal and the human body can tolerate titanium in large doses [22].
Cobalt (Co)	Its function limits the role of vitamin B12 [16], by diminishing the adsorption of Fe in the blood stream [23]. The normal concentration of Co in human fluids is 1.5 mg/L.
Chromium (Cr)	It causes ulceration and central nerve system disorder [23]. The maximal concentration in the blood stream should be 28 mg/L. Its compounds are adsorbed only after oral ingestion. Cr (III) is usually deposited in reticular systems within the cell, while Cr (IV) is capable to penetrate the cellular membrane in both directions [21].
Aluminum (Al)	It provokes epileptic episodes and Alzheimer [23]. The maximal concentration in the blood stream should be 30 mg/L.
Vanadium (V)	It is very toxic in its elementary state [23], therefore the maximum concentration should not exceed 0.5 µg/L.
Molybdenum (Mo)	It is an essential element used by specific enzymes, thus is easily adsorbed trough the intestines and its normal concentration in the blood stream should be 1–3 ppm. It is very toxic and sometimes lethal in large doses, regular symptoms being diarrhea, coma, heart failure and inhibitor for some essential enzymes. Also, large concentrations of Mo can interfere with Ca and P metabolism [21].

**Table 2:** Reagents for preparing the updated simulated body fluid [24] (reprinted from ref. [24] with permission from Elsevier).

Reagent	Amount for 1 L of SBF
Sodium chloride	8.035 g
Sodium bicarbonate	0.355 g
Potassium chloride	0.225 g
Potassium phosphate dibasic trihydrate	0.231 g
Magnesium chloride hexahydrate	0.311 g
1 M hydrochloric acid	39 mL
Calcium chloride	0.292 g
Sodium sulfate	0.072 g
Tris(hydroxymethyl) amino methane	6.118 g

**Table 3:** Metallic concentrations of the samples.

Sample	Ti (mg/L)	Co (mg/L)	Cr (mg/L)	Mo (mg/L)	Si (mg/L)	Fe (mg/L)	Mn(mg/L)	Ni (mg/L)
1	12.971	11.401	4.475	0.161	0.287	2.606	0.116	2.656
2	10.176	1.421	0.597	0.011	0.416	7.271	0.038	74.935
3	14.121	0.145	0.052	0.009	0.076	12.544	0.041	36.134

**Table 4:** Initial metallic sample characteristics.

Sample	Surface, (mm^2^)	Initial Weight, (g)	Initial State	
1	1427.93	7.1576	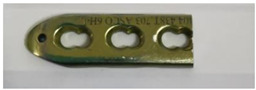	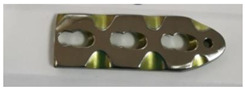
2	1256.75	8.0665	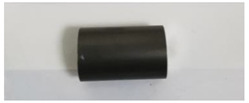	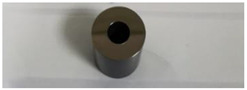
3	1101.29	3.9136	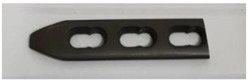	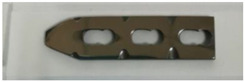

**Table 5:** Corrosion rate and ion release rate.

Sample	Corrosion Rate (g/(mm^2^ × Week))	Corrosion Rate (mm/Year)	Ion Release ((mg/L)/(mm^2^ × Week))					
			Co	Ni	Ti	Cr	Mo	Mn
1	4.3 × 10^−5^	0.315	1.89 × 10^−6^	76.6 × 10^−6^	1.8 × 10^−4^	0.2 × 10^−4^	0.1 × 10^−4^	0.2%
2	4.9 × 10^−5^	0.308	14.8 × 10^−6^	3 × 10^−6^	2.6 × 10^−4^	1.7 × 10^−4^	5.2 × 10^−4^	1.3%
3	5.6 × 10^−5^	0.259	11.2 × 10^−6^	6.7 × 10^−6^	2.3 × 10^−4^	34.2 × 10^−4^	7.6 × 10^−4^	1.6%

## Data Availability

Not applicable.

## References

[1] Dimah M.K., Albeza F.D., Borras V.A., Muñoz A.I. (2012). Study of bio-tribocorrosion of titanium biomedical alloys in simulated body fluids by electrochemical techniques. Wear.

[2] Mischler S., Munoz A.I. (2013). Wear of CoCrMo alloys used in metal-on-metal hip joints: A tribocorrosion appraisal. Wear.

[3] Diomidis N., Mischler S., More N.S., Manish R. (2012). Tribo-electrochemical characterization of metallic biomaterials for total joint replacement. Acta Biomater..

[4] Baino F., Yamaguchi S. (2020). The use of simulated body fluid (SBF) for assessing material bioactivity in the context of tissue engineering: Review and challenges. Biomimetics.

[5] Petkovic D.S., Mandrino D., Sarler B., Horky J., Ojdanic A., Zehetbauer M.J., Orlov D. (2020). Surface analysis of biodegradable Mg-alloys after immersion in simulated body fluid. Materials.

[6] Bidhendi H.R.A., Pouranvari M. (2012). Corrosion study o metallic biomaterials in simulated body fluid. Metall. Mater. Eng..

[7] Mohan P., Rajak D.K., Pruncu C.I., Behera A., Amigo-Borras V., Elshalakany A.B. (2021). Influence of β-phase stability in elemental blended Ti-Mo. And Ti-Mo.-Zr alloys,-phase stability in elemental blended Ti-Mo. And Ti-Mo.-Zr alloys. Micron.

[8] Baltatu M.S., Tugui C.A., Perju M.C., Benchea M., Spataru M.C., Sandu A.V., Vizureanu P. (2019). Biocompatible titanium alloys used in medical applications. Rev. Chem..

[9] Mirea R., Ceatra L., Cucuruz A.T., Ene R., Popescu E., Biris I., Cretu M. (2020). Advanced experimental investigation of used metallic biomaterials. Rom. J. Mater..

[10] Park J.B., Bronzino J.D. (2002). Biomaterials—Principles and Applications.

[11] Yaszemski M.J. (2004). Biomaterials in Orthopedics.

[12] Türkan U., Öztürk O., Eroğlu A.E. (2006). Metal ion release from TiN coated CoCrMo orthopedic implant material. Surf. Coat. Technol..

[13] Hanawa T., Hiromoto S., Asami K. (2001). Characterization of the surface oxide film of a Co–Cr–Mo. alloy after being located in quasi-biological environments using XPS. Appl. Surf. Sci..

[14] Totea G., Ionita D., Demetrescu I. (2014). ICP-MS determinations in sustaining corrosion data of 316 stainless steel in bio-liquids. UPB Sci. Bull. B Chem. Mater. Sci..

[15] Lee T., Eshaan M., Rajaraman S., Manivasagam G., Singh A.K., Lee C.S. (2015). Tribological and corrosion behaviors of warm-and hot-rolled Ti-13Nb-13Zr alloys in simulated body fluid conditions. Int. J. Nanomed..

[16] Yang X., Hutchinson C. (2016). Corrosion-wear of β-Ti alloy TMZF (Ti-12Mo-6Zr-2Fe) in simulated body fluid. Acta Biomater..

[17] Tocker S.M., Gerstein G., Maier H.J., Canadinc D. (2018). Effects of microstructural mechanisms on the localized oxidation behavior of NiTi shape memory alloys in simulated body fluid. J. Mater. Sci..

[18] Samad H.A., Rashid R.A. (2017). Characterization study of industrial waste glass as starting material in development of bioactive materials. J. Fund. Appl. Sci..

[19] (2017). Speedwave Xpert Microwave Digestion System, User Manual, Ver. 2.3, Appl. Note Microwave Digestion of Cr/Mo/Ni Alloy. https://www.sysmex.nl/fileadmin/media/f102/MLS/2018_03_28-Broschuere-Mikrowelle_EN_final.pdf.

[20] Popa M., Demetrescu I., Vasilescu E., Drob P., Ionita D., Vasilescu C. (2009). Corrosion Resistance of Some Thermo-mechanically Treated Titanium Bioalloys Depending on pH of Ringer Solution. Rev. Roum. Chim..

[21] Bhat S.V. (2002). Biomaterials.

[22] https://www.lenntech.com/periodic/elements/ti.htm.

[23] Aksakal B., Yildirim Ö.S., Gul H. (2004). Metallurgical failure analysis of various implant materials used in orthopedic applications. J. Fail. Anal. Prev..

[24] Marques M.R.C., Loebenberg R., Almukainzi M. (2011). Simulated Biological Fluids with Possible Application in DissolutionTesting. Dissolution Technol..

[25] Nica M., Cretu B., Ene D., Antoniac I., Gheorghita D., Ene R. (2020). Failure Analysis of Retrieved Osteosynthesis Implants. Materials.

